# Deep Brain Stimulation of the Antero-Medial Globus Pallidus Interna for Tourette Syndrome

**DOI:** 10.1371/journal.pone.0104926

**Published:** 2014-08-19

**Authors:** Perminder S. Sachdev, Adith Mohan, Elisabeth Cannon, John D. Crawford, Paul Silberstein, Raymond Cook, Terrence Coyne, Peter A. Silburn

**Affiliations:** 1 Neuropsychiatric Institute, Prince of Wales Hospital, Randwick, NSW, Australia; 2 Centre for Healthy Brain Ageing, School of Psychiatry, The University of New South Wales, Sydney, Australia; 3 North Shore Private Hospital, St. Leonards, Sydney, NSW, Australia; 4 Centre for Clinical Research, University of Queensland, Herston, QLD, Australia; 5 St. Andrew's War Memorial Hospital, Spring Hill, QLD, Australia; University of California at San Francisco, United States of America

## Abstract

**Background:**

We have previously reported the results of Deep Brain Stimulation (DBS) of the antero-medial globus pallidus interna (GPi) for severe Tourette Syndrome (TS) in 11 patients. We extend this case series to 17 patients and a longer follow-up to a maximum of 46 months.

**Methods:**

17 patients (14 male; mean age 29.1 years, range 17–51 years) with severe and medically intractable TS were implanted with Medtronic quadripolar electrodes bilaterally in the antero-medial GPi. The primary outcome measure was the Yale Global Tic Severity Scale (YGTSS). Secondary outcome measures included the Yale-Brown Obsessive Compulsive Scale, Hamilton Depression Rating Scale, Gilles de la Tourette Quality of Life Scale and Global Assessment of Functioning. Follow up was at one month, three months and finally at a mean 24.1 months (range 8–46 months) following surgery.

**Results:**

Overall, there was a 48.3% reduction in motor tics and a 41.3% reduction in phonic tics at one month, and this improvement was maintained at final follow-up. 12 out of 17 (70.6%) patients had a>50% reduction in YGTSS score at final follow up. Only 8 patients required ongoing pharmacotherapy for tics post-surgery. Patients improved significantly on all secondary measures. Adverse consequences included lead breakage in 4 patients, infection (1), transient anxiety (2), dizziness (1), poor balance (1) and worsening of stuttering (1).

**Conclusions:**

This case series provides further support that antero-medial GPi DBS is an effective and well tolerated treatment for a subgroup of severe TS, with benefits sustained up to 4 years.

## Introduction

The last decade has seen an increasing number of reports of deep brain stimulation (DBS) for the treatment of medically-intractable Tourette Syndrome (TS), with nearly 100 cases having been reported in the published literature [1]–[4]. Although nine different brain targets have been reported [5], the most commonly used targets have been the centromedian-parafascicular and ventralis oralis complex of the thalamus [6], and the globus pallidus interna (GPi) [7].

We recently reported an open case series of 11 TS patients who were treated with DBS of the antero-medial GPi and were followed up for a mean 14 months, with the longest follow-up periods being 30 months [8]. The study showed that the acute benefit of DBS was maintained over this follow-up period, and adverse effects were encountered in only a few patients. Since TS is a chronic disorder, the long-term outcome over many years is important to determine. In the longest follow-up reported so far in the literature, 15 patients with thalamic stimulation were followed up for 5–6 years and three patients for 3–4 years. While the overall improvement was maintained, some notable findings were that four patients had a worsening of their obsessive compulsive symptoms and two requested device removal more than 5 years after implantation. Issues with non-compliance and repeated infections were also noted, highlighting the importance of longer-term follow-up in these cases.

We report the results of an extended series of TS patients with a longer follow-up to complement our previous report. The 17 patients being reported are inclusive of the 11 reported previously.

## Methods

*Ethics statement*: All information collected was entered into a dedicated, purpose designed data registry. The data registry is regarded as an audit activity that does not require patient consent. The Uniting Care Health Human Research Ethics Committee (Ref. 0813) deemed that a formal ethical review was not required. This follow-up study was undertaken at St Andrew's War Memorial Hospital, Spring Hill, QLD 4000, and at the North Shore Private Hospital, St Leonards, NSW 2065, Australia.

Patients with severe, disabling and medically resistant TS were recruited from the Movement Disorders Clinic of St Andrew's War Memorial Hospital, Brisbane (n = 15) and Neuropsychiatric Institute, Sydney (n = 2). Criteria for selection for DBS have been described previously (5). A summary of individual patient characteristics is shown in Table 1.

**Table 1:** Summary of individual patient characteristics.

No.	Age	Sex	Consent	Electrode location	Follow up (mths)	Severity of illness	No. drugs tried before DBS	OCD	MD	Attentional Problems
1	27	M	Y	Bilat Gpi	28	Severe	4	Y	Y	N
2	19	F	Y	Bilat Gpi + Nac	46	very severe	6	Y	Y	Y
3	35	F	Y	Bilat Gpi	30	severe	4	Y	Y	N
4	26	F	Y	Bilat Gpi	36	severe	5	Y	N	N
5	34	M	Y	Bilat Gpi + Nac	41	Severe	4^1^	Y	Y	N
6	28	M	Y	Bilat Gpi	22	Severe	6	Y	Y	Y
7	23	M	Y	Bilat Gpi	30	Severe	3	N	N	N
8	31	M	Y	Bilat Gpi	22	Severe	4	N	N	N
9	28	M	Y	Bilat Gpi	35	Severe	4	Y	Y	N
10	39	M	Y	Bilat Gpi	20	Severe	6	Y	N	N
11	51	M	Y	Bilat Gpi	23	Severe	3	N	N	N
12	42	M	Y	Bilat Gpi	12	Severe	>3	N	N	N
13	17	M	Y	Bilat Gpi	16	very severe	6	N	Y	Y
14	19	M	Y	Bilat Gpi	9	Severe	>3	Y	N	N
15	18	M	Y	Bilat Gpi	8	Severe	>3	Y	Y	N
16	17	M	Y	Bilat Gpi	18	very severe	6	Y	N	Y
17	30	M	Y	Bilat Gpi	13	Severe	6	Y	N	N

1 including botulinum toxin.

Prior to surgery, each patient completed a series of questionnaires, including the Yale Global Tic Severity Scale (YGTSS) [9] which was the primary outcome measure. Secondary outcome measures included the Yale-Brown obsessive-compulsive Scale (Y-BOCS) [10], Hamilton Depression Rating Scale (HDRS) [11], Gilles de la Tourette Quality of Life Scale (GTS-QOL) [12] and the Global Assessment of Functioning (GAF) scale [13].

Surgeries were performed by a team comprising a psychiatrist, a neurologist and neurosurgeon, and were performed solely on the basis of clinical indication. The assessments for this paper were performed by psychiatrists who were independent of the decision to perform the surgery. Consent was obtained by the surgeon for the surgical procedure. For the rating scales and the assessment, written consent was obtained from the patient. In the case of two patients who were <18 years at the time of surgery, the consent was obtained in the presence of the mother in each case.

A Medtronic multi-programmable quadripolar deep brain stimulation system was implanted under general anesthetic using a stereotaxic procedure as described previously [5]. Each patient received either the Soletra system (Model 7426) which has a separate battery for each side, or the Activa PC/RC device (Model 37601/37612) (Medtronic, Inc, Minneapolis MN, USA) which has a single battery for both sides. Medtronic leads 3389 in cases 1–15, and 3387 in cases 16 and 17, were placed with active contacts spanning the GPi/GPe, the choice of leads reflecting the surgeon's preference.

The anatomical target was a modification of the posteroventral pallidal (PVP) target popularized by Laitinen [14]. We have described this target previously [15].

**Figure 1:**
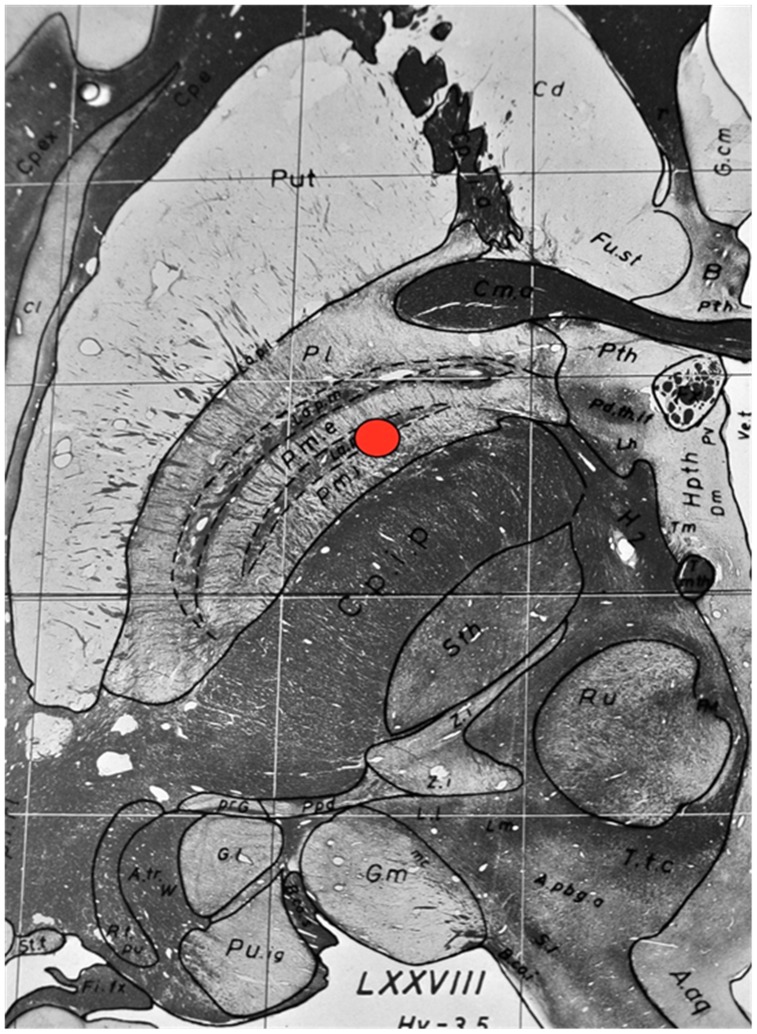
An image from the “Atlas for Stereotaxy of the Human Brain”: Schaltenbrand and Wahren, Plate 54 [25] showing a the stimulation target as red marker overlying the anteromedial pallidum on an axial brain slice at 3.5 mm below the AC/PC line.

In reference to the anterior commissure/posterior commissure (AC/PC) line, the PVP target was 2 mm anterior to the midpoint of the AC/PC line, between 18–21 mm lateral to the midline and 3–5 mm below the AC/PC plane. The antero-medial pallidal target used in this series was approximately 6 mm anterior to the AC/PC midpoint and 14–17 mm lateral to the midline at the same depth as the PVP target 3–5 mmm below the AC/PC plane. With reference to the PVP target, it is approximately 4 mm anterior and 4 mm medial to this target. Indirect calculations based on the AC/PC plane or line are however only a guide as the use of 3T MRI/CT fusion is standard practice owing to the inherent variability in brain dimensions, and the real anatomical target is seen in Figure 1 as the red marker in relation to the internal pallidum and surrounding structures. Once the target is defined anatomically, microelectrode techniques are used mainly to define the inferior pallidal border. The inferior electrode (0) is placed 2 mm above the pallidal base with the remaining 3 electrodes spaced and angled 50–70 degrees in the sagittal plane with the plane of the base ring (CRW)/arc parallel to the AC/PC plane. The coronal angle is wide, varying 15–25 degrees from the midpoint of the arc. This allows for a DBS electrode trajectory from the above-defined target passing through the internal and external GPi, the GPe/GPi raphe into the external pallidum.

The Medtronic impulse generator was placed in the subclavicular region. A post-operative brain CT scan was undertaken and fused to the pre-operative MRI image to confirm correct lead positioning.

DBS stimulation was commenced in the immediate post-operative period when the patient had recovered from the propofol anesthetic and all patients were closely monitored. Initial stimulation parameters were bilateral 1.0 V, pulse width 90 µs and frequency 130 Hz with unipolar activation of the contacts most commonly 0, 1 or 2 and rarely 3. Stimulation parameters were adjusted at follow up visits depending on residual tic severity and presence of side effects. The most commonly used increments were 0.5 V every second day. The mean stimulation parameters at final follow up were amplitude 4.14 V (SD 0.63), pulse width 95.2 µs (SD 21.8) and frequency 139.4 Hz (SD 19.5). The final stimulation parameters for each of the patients are documented in Table S1 in File S1.

Patients were followed up weekly for 1 month, monthly for 3 months and then 3 monthly. Stimulation parameters were adjusted as required over the course of follow up. Rating scales were repeated at one month after surgery and at final follow up (mean: 23.4 months, range: 8–46 months). At these time points, information was obtained on any adverse effects. Assessments were performed by three authors (PSS, EC and AM) independently of the surgical teams (PSilburn, TC, PSilberstein, RC).

Data were analyzed using IBM SPSS Statistics 21 (SPSS, IBM Corp, Armonk NY, USA). The non-parametric Friedman test was used to test for the statistical significance of variation of measures across the 4 time points. Paired comparisons between measures at the pre-DBS and 3 post-DBS time points were performed using the Wilcoxon Signed-Ranks Tests. Relationships between treatment response, as evaluated by changes in the primary outcome measures derived from the YGTSS, and a number of potential predictors were evaluated. Spearman's rank-order correlations were used for continuous predictors and the Mann-Whitney U test for binary predictors. Due to multiple tests, an alpha of 0.01 was considered statistically significant.

## Results

### Treatment response

Out of 17, 16 (94%) patients reported having a positive response to DBS, with self-perceived reduction in tic number, severity and frequency. Defining response as >50% reduction in the Total TYGTSS score, 12 out of 17 (70.6%) patients responded to DBS, although three others also had a meaningful improvement at final follow up, as evidenced by changes in GTS-QOL and GAF scores (Table S3 in File S1).

**Figure 2:**
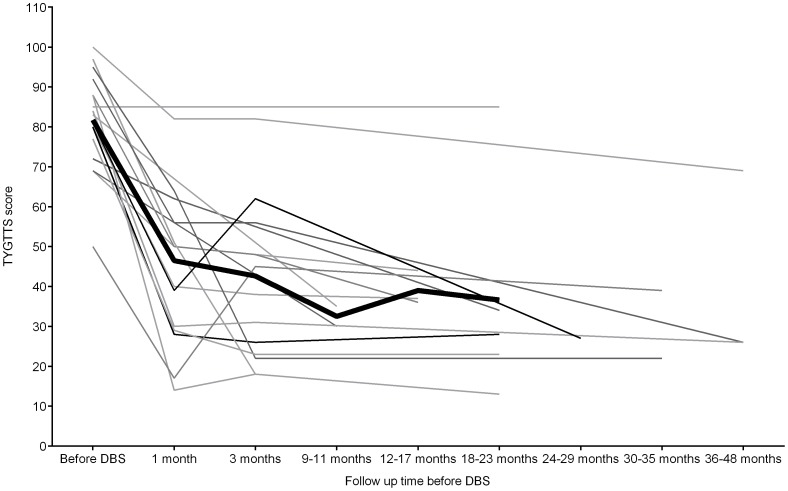
Change in Total Yale Global Tic Severity Score (TYGTTS) at different time points pre and post-DBS. Bold line indicates the mean of the scores. Mean scores were only displayed when more than 3 data points were present. There was a downward trend or no change in the mean scores after 23 months.

Significant improvements were noted in all four components of the YGTSS scores, as was the case with secondary outcome measures. At final follow up 47.8% reduction in motor tics and 51.5% reduction in phonic tics were noted. In the 12 patients considered ‘responders’, there was greater than 50% reduction in both motor (72.5%) and phonic (60.4%) tics at final follow up. Overall, there was a reduction in the mean TYGTSS score from 81.18 before surgery to 37.12 at final follow-up representing a 54.3% reduction in total tic severity (Z = 3.52, p≤0.001) (see Table 2 and Tables S2 and S3 in File S1). The mean TYGTSS scores, and those for individual patients at each of the four assessment occasions, are presented in Figure 2.

**Table 2:** Rating scale scores for 17 patients with Tourette syndrome with deep brain stimulation of the globus pallidus interna.

	BL (N = 17)	1 month (N = 15)	3 months (N = 14)	Final (N = 17)	Friedman test for overall effect of time (N = 14)	Wilcoxon Signed Ranks tests for paired comparisons, Z (p)		
	Mean (SD)	Mean (SD)	Mean (SD)	Mean (SD)	Chi Squared (d.f. = 3) (p)	1 month versus BL	3 months versus BL	Final versus BL
**M**	21.53 (2.58)	11.13 (6.41)	10.50 (6.54)	11.24 (5.98)	25.8 (.000)	3.30 (.000)	3.18 (.000)	3.52 (.000)
**V**	17.59 (4.87)	10.33^a^ (5.86)	8.00 (6.19)	8.53^a^ (5.77)	26.5 (.000)	3.24 (.000)	3.19 (.000)	3.52 (.000)
**TTSS**	39.12 (6.99)	21.47 (10.52)	18.43 (11.24)	19.76 (10.65)	27.2 (.000)	3.30 (.000)	3.19 (.000)	3.52 (.000)
**TYGTSS**	81.18 (12.32)	45.80 (20.16)	41.29 (20.92)	37.12 (19.53)	28.7 (.000)	3.30 (.000)	3.18 (.000)	3.52 (.000)
**YBOCS**	13.88 (11.73)	6.80 (9.14)	5.29 (8.52)	5.29 (7.07)	14.6 (.001)	2.55 (.008)	2.55 (.008)	2.94 (.001)
**HDRS**	15.35 (7.57)	6.60 (4.55)	5.07^b^ (4.20)	8.00^b^ (5.60)	15.8 (.001)	2.96 (.002)	2.96 (.002)	3.03 (.001)
**GAF**	50.00 (14.14)	76.00 (14.04)	75.71 (12.84)	72.12 (14.60)	27.7 (.000)	3.29 (.000)	3.20 (.000)	3.45 (.000)
**GTS_QOL**	40.88 (20.48)	72.67 (15.57)	76.43^a^ (16.81)	66.47^a^ (17.03)	32.5 (.000)	3.45 (.000)	3.32 (.000)	3.53 (.000)

M =  motor tics; V =  vocal/phonic tics; TTSS =  Total Tic Severity Scale; TYGTSS =  Total Yale Global Tic Severity Scale; YBOCS =  Yale-Brown Obsessive-Compulsive Scale; HDRS =  Hamilton Depression Rating Scale; GAF =  Global Assessment of Functioning; GTS-QOL =  Gilles de la Tourette Syndrome Quality of Life Scale.

Compared with changes from baseline, variation in mean scores for the four YGTSS measures across the last three occasions was much smaller, with none of the differences between the post DBS occasions being statistically significant. The decrease in the phonic tic scale at 1 month to final assessment (Z = 1.99, p = 0.049; see Table 2) was not statistically significant after correction for multiple testing (α = 0.01).

Of the 17 patients in the study, 11 (64.7%) patients reported clinically significant obsessive compulsive symptoms prior to DBS on the Y-BOCS, which decreased from a mean score of 13.88 at pretest to 5.29 at the final assessment (Z = 2.94, p = 0.001). There was also a reduction in mean HDRS scores from 15.35 at pretest to 8.00 at the final assessment (Z = 3.03, p = 0.001). There were significant improvements between pretest and final scores in the GTS-QOL, from 40.88 to 66.47 (Z = −3.53, p<0.001), and in GAF scale, from 50.0 to 72.12 (Z = 3.45, p<0.001).

As with the YGTSS measures, variation across the three post-DBS occasions for the secondary measures were much smaller than between these and the pretest scores. The HDRS score increased slightly from the 3 months value of 5.07 to the final assessment score of 8.00 (Z = 2.58, p = 0.007), although the final value was still well below the pre-DBS score of 15.35. There was also a relatively small decline in the Quality of Life score from the 3 months value of 76.43 to the final assessment mean score of 66.43 (Z = 2.51, p = 0.014), although, again, the final value was still well below the pre-DBS score of 40.88.

### Outlying patients

Only two patients failed to gain clinically significant benefit from DBS stimulation (Patients 9 and 11). The first of these, patient 9, was in fact a treatment responder at the end of the first wave follow up period, but had a device battery failure associated with severe tic recurrence and a relapse of pre-DBS pattern of substance abuse. The consequent family discord culminated in an acute psychiatric admission. This patient had his device explanted in the aftermath of these events.

The second patient (no. 11), reported previously [5], suffered a worsening of his tics and somatic symptoms with the stimulation and elected to have it switched off at 3 months. At 51 years of age, he was the oldest patient in the cohort, and had a chronically severe condition.

### Predictors of treatment response

No significant relationships were found between treatment response, as measured by change in TYGTSS score, and a number of potential predictors, which included patient characteristics shown in Table 1, and pre-DBS scores on YBOCS, HDRS, GAF and GTS_QOL. Whether the tics were primarily motor or phonic was also not a predictor of response. This was true even when using the liberal type-one error rate of 0.10.

### Adverse effects

No procedure related complications were noted. The main device related adverse effect was cable breakage in 4 patients, due to a motor vehicle accident in one, an inadvertent self-inflicted blow to the chest as a complex motor tic in another, a self-injurious tic in the third, and no obvious cause in the fourth. One patient developed an infection around the leads in the neck 3 months after surgery and required bilateral lead replacement. In 3 patients, hardware malfunction resulted in interruption to stimulation during which time worsening in tic severity was the main adverse effect, with subsequent improvement once stimulation was re-established. Relapse of substance abuse and subsequent device explantation in patient 9 have been previously noted. The psychological distress experienced by patients upon cessation of stimulation following a therapeutic response, has been documented by other investigators [12].

Side effects related to stimulation itself were mostly temporary, and attenuated with adjustment of stimulation parameters. These included transient anxiety (2 patients), agitation upon stimulation of most caudal contacts (2 patients), dizziness (1 patient), poor balance (1 patient) and worsening of pre-existing stuttering (1 patient). The phenomenology of the stuttering in this last patient was of significance in that it he appeared to suffer intermittent speech arrest, manifesting as a stutter that improved to pre-operative levels with stimulation reduction. Speech dysfluency has been previously been reported as a stimulation related consequence of both pallidal [17] and subthalamic nucleus DBS [18]. Patient 11 reported worsening in tic severity with stimulation and eventually elected to have his stimulator switched off.

## Discussion

We present extended follow up data from a case series of patients who underwent DBS for intractable TS. In addition to the 11 patients described previously [8], we have described data on 6 more patients, making this amongst the largest series of TS patients treated with DBS of the antero-medial globus pallidus interna (GPi) from one centre. The extended duration of follow up makes it possible to comment on the time course of symptom remission in these patients and the long-term outcome of DBS.

Using a>50% reduction in the TYGTSS score as the criterion for treatment response, 12 out of 17 (70.6%) patients responded to DBS of the antero medial GPi. This was a cohort of severely to very severely affected patients with Tourette syndrome, all of whom had failed treatment with at least 3 or more pharmacological therapies. In considering the patients reported in our previous paper first [8], of the 6 patients classed as responders at a mean of 14 months after surgery (range  = 4–30 months), 5 remained responders, with TYGTSS scores at second wave follow up closely resembling their earlier final follow up scores. All 5 had attained responder status between 1 and 3 months follow up. 1 patient classed as non responder in the first report (patient 1) had in fact achieved treatment responsiveness at 1 month, but had a subsequent worsening, although remaining clinically much improved relative to baseline. This patient had re-attained responder status by the time of second wave follow up as reported in this paper.

Only 1 new responder was added to this data set from the initial cohort of 11 patients (patient 5), reaching >50% TYGTSS reduction between 26 and 41 months. It is possible that this represents a propensity to delayed treatment response in a minority of these patients, though this is difficult to extrapolate from just one patient.

On the whole, our data would suggest that the time to respond to antero medial GPi DBS in this cohort is short, being between 1–3 months, and that symptomatic gains, once achieved, remain stable over time. The percentage reductions in tics were remarkably similar in both first and second waves of follow up, for both motor (48% and 47.8%) as well as phonic (54.5% and 51.5%) tics respectively. The time difference between the mean follow up durations for these two waves was about 10 months. This stability of effect on tic reduction in TS with DBS has been noted by another group as well [19], with patients continuing to show significant reduction in tic severity, and consistently requiring less medication or treatment of their TS as well as associated comorbidities over 6 years of follow up. Similarly, in long term follow up data at 3–6 years reported on by Kennedy and colleagues [20] for DBS in depression, short and long term response, and remission rates remained stable over time. Holtzheimer and colleagues made the important observation that none of their depressed patients who remitted on being treated with DBS of the subcallosal cingulate, suffered a spontaneous relapse over 2 years of follow up [16]. In a review of DBS in OCD undertaken across four centers in the United States and Europe [21], symptomatic improvements were noted to occur by 3 months on average, and to remain stable over 3 to 36 months of follow. Taken together, converging evidence appears to indicate that in DBS for refractory neuropsychiatric disorders, relatively early treatment response is to be expected in most, and in those that do respond, treatment benefit is maintained over several years.

Most importantly, stable symptom improvement in our cohort was translated in the majority of patients into substantial improvements in vocational functioning, as well as in the patient's relationships with family and friends. The wide ranging impact of TS on sufferers is well known, and although almost all our patients continued to display clinically relevant symptomatology, it is notable that the degree of improvement noted with DBS in these patients leads to significant gains in their day to day lives. Modest gains noted on symptom rating scores, often translate into greater functional gains, reflective of the high pretreatment severity of illness in such DBS cohorts. This is reflected more accurately in the improvements noted in the quality of life, and functional status ratings of this cohort (GTS-QOL and GAF respectively) for responders and the majority of non responders alike. All but the two patients described as outliers in the results, elected to continue with stimulation in light of these improvements.

It is worthwhile noting that all 6 patients recruited into our study between the first and current time points were responders. Better outcomes with patients enrolled later in DBS trials have been conceptualized as being a ‘learning effect’. This has been attributed to refinements in targeting, advances in surgical technique and better patient management including programming protocols, with similar improvements noted with patients [22] recruited over time in DBS for other movement disorders as well. Even though our surgical target remained the antero medial GPi, given inter-individual neuroanatomical variability, and small target size in TS, relative to more conventional targets in DBS for movement disorders such as PD, future work focusing on more detailed comparisons of responders and non responders in terms of final lead location within GPi, and computer modeling to predict the field of stimulation would be instructive.

The importance of long term follow data in this patient population is beginning to become increasingly apparent. In a recent report, follow up data were reported on for 15 patients at 5–6 years and 3 patients at 3–4 years who underwent thalamic DBS for refractory TS [19]. In this, the authors make note of the emergence of a number of issues over time, chief amongst these being infections, treatment discontinuation, and a concerning lack of consensus between clinician's and patient's perspectives on the extent of improvement. In our long term data by contrast, we noted consistency across clinician and patient ratings over time, and lower rates of treatment discontinuation. The adverse effect profile has differed in DBS for TS depending on the target chosen [23], which in turn affect patient compliance. The antero medial GPi in this context, appears to be a relatively safe, with comparable efficacy to thalamic targets for TS DBS.

### Limitations

We, along with others [24], have previously commented on the limitations of an open study without placebo control and with non-blinded assessments. However, the long-term follow-up data further support the argument against this being a placebo response. This case series is still limited by the lack of a comparison site of stimulation, making it difficult to argue that this is indeed the optimal implantation site. Moreover, more refined analysis in terms of simulation of the anatomical region actually stimulated, and neuroimaging to understand likely mechanisms of response have not been possible in this study and will serve as objectives for future work.

## Supporting Information

File S1 **Table S1)** Stimulation parameters for the patients at time of final assessment. **Table S2)** Individual patient Total Yale Global Tic Severity Scale scores before DBS and at final follow up. **Table S3)** Individual patient Yale-Brown Obsessive Compulsive Scale (YBOCS), Hamilton Depression Rating Scale (HDRS), and Gilles de la Tourette Quality of Life Scale (GTS-QOL) scores before DBS and at final follow up.
(DOCX)
