# Supplementary material for: Deep Brain Stimulation of the Antero-Medial Globus Pallidus Interna for Tourette Syndrome
Source: PLoS One. 2014 Aug 19;9(8):e104926. doi: 10.1371/journal.pone.0104926 (PMC4138156; doi:10.1371/journal.pone.0104926)
Supplement: File S1 — Table S1) Stimulation parameters for the patients at time of final assessment. Table S2) Individual patient Total Yale Global Tic Severity Scale scores before DBS and at final follow up. Table S3) Individual patient Yale-Brown Obsessive Compulsive Scale (YBOCS), Hamilton Depression Rating Scale (HDRS), and Gilles de la Tourette Quality of Life Scale (GTS-QOL) scores before DBS and at final follow up. (DOCX) [file pone.0104926.s001.docx]

**Table S1.** Stimulation parameters for the patients at time of final assessment.

| **Patient No.** | **System** | **Wave Amplitude (V)** | **Pulse Width (ms)** | **Frequency (Hz)** |
| --- | --- | --- | --- | --- |
| 1 | Activa | 4.6 | 90 | 160 |
| 2 | Soletra | 4 | 60 | 160 |
| 3 | Activa | 4.2 | 120 | 130 |
| 4 | Soletra | 3.6 | 120 | 100 |
| 5 | Soletra | 3.3 | 90 | 160 |
| 6 | Activa | 5 | 120 | 160 |
| 7 | Activa | 5 | 90 | 160 |
| 8 | Activa | 4.6 | 90 | 130 |
| 9 | Activa | 4 | 90 | 160 |
| 10 | Soletra | 3.7 | 60 | 130 |
| 11 | Soletra | 3 | 60 | 160 |
| 12 | Activa | 4.8 | 120 | 130 |
| 13 | Activa | 3.8 | 90 | 130 |
| 14 | Activa | 5 | 90 | 110 |
| 15 | Activa | 3.6 | 120 | 130 |
| 16 | Activa | 3.6 | 90 | 130 |
| 17 | Activa | 4.5 | 120 | 130 |

**Table S2.** Individual patient Total Yale Global Tic Severity Scale scores before DBS and at final follow up.

| **Patient No.** | **Final follow up (months)** | **M**  **Pre** | **M**  **Post** | **V**  **pre** | **V**  **post** | **TTSS**  **Pre** | **TTSS**  **Post** | **TYGTSS**  **Pre** | **TYGTSS**  **Post** |
| --- | --- | --- | --- | --- | --- | --- | --- | --- | --- |
| 1 | 28 | 21 | 12 | 10 | 5 | 31 | 17 | 81 | 27 |
| 2 | 46 | 25 | 18 | 25 | 11 | 50 | 29 | 100 | 69 |
| 3 | 30 | 18 | 12 | 12 | 7 | 30 | 19 | 50 | 39 |
| 4 | 36 | 22 | 10 | 22 | 6 | 44 | 16 | 84 | 26 |
| 5 | 41 | 22 | 7 | 20 | 9 | 42 | 16 | 92 | 26 |
| 6 | 22 | 21 | 9 | 16 | 4 | 37 | 13 | 77 | 23 |
| 7 | 30 | 25 | 8 | 20 | 4 | 45 | 12 | 95 | 22 |
| 8 | 22 | 24 | 3 | 14 | 0 | 38 | 3 | 88 | 13 |
| 9 | 35 | 22 | 20 | 25 | 20 | 47 | 40 | 97 | 80 |
| 10 | 20 | 22 | 6 | 18 | 2 | 40 | 8 | 80 | 28 |
| 11 | 23 | 24 | 24 | 16 | 16 | 40 | 40 | 75 | 75 |
| 12 | 12 | 20 | 4 | 18 | 12 | 38 | 16 | 88 | 36 |
| 13 | 16 | 20 | 5 | 20 | 12 | 40 | 17 | 80 | 37 |
| 14 | 9 | 15 | 10 | 14 | 10 | 29 | 20 | 69 | 30 |
| 15 | 8 | 21 | 19 | 22 | 16 | 43 | 35 | 83 | 35 |
| 16 | 6 | 24 | 11 | 8 | 0 | 32 | 11 | 72 | 21 |
| 17 | 13 | 20 | 13 | 19 | 11 | 39 | 24 | 69 | 44 |

M = motor; V – vocal tics; TTSS = M+V; TYGTSS = TTSS+impairment

**Table S3.** Individual patient Yale-Brown Obsessive Compulsive Scale (YBOCS), Hamilton Depression Rating Scale (HDRS), and Gilles de la Tourette Quality of Life Scale (GTS-QOL) scores before DBS and at final follow up.

| **Patient No.** | **YBOCS**  **Pre** | **YBOCS**  **Post** | **HDRS**  **Pre** | **HDRS**  **Post** | **GAF**  **Pre** | **GAF**  **Post** | **GTS-QOL**  **Pre** | **GTS-QOL Post** |
| --- | --- | --- | --- | --- | --- | --- | --- | --- |
| 1 | 25 | 0 | 26 | 5 | 50 | 90 | 40 | 70 |
| 2 | 30 | 18 | 16 | 17 | 20 | 50 | 20 | 40 |
| 3 | 22 | 12 | 17 | 11 | 50 | 80 | 30 | 60 |
| 4 | 19 | 0 | 17 | 6 | 60 | 80 | 70 | 70 |
| 5 | 31 | 16 | 26 | 11 | 50 | 80 | 65 | 70 |
| 6 | 27 | 14 | 15 | 13 | 40 | 70 | 20 | 70 |
| 7 | 0 | 0 | 9 | 0 | 50 | 90 | 15 | 90 |
| 8 | 0 | 0 | 4 | 5 | 80 | 90 | 70 | 90 |
| 9 | 0 | 0 | 26 | 12 | 30 | 50 | 15 | 70 |
| 10 | 20 | 4 | 18 | 6 | 40 | 80 | 50 | 80 |
| 11 | 0 | 0 | 7 | 5 | 50 | 50 | 35 | 45 |
| 12 | 0 | 0 | 12 | 11 | 50 | 80 | 30 | 60 |
| 13 | 11 | 0 | 10 | 18 | 60 | 60 | 40 | 50 |
| 14 | 13 | 1 | 16 | 0 | 50 | 80 | 40 | 80 |
| 15 | 14 | 7 | 27 | 12 | 40 | 60 | 20 | 30 |
| 16 | 1 | 1 | 4 | 1 | 60 | 90 | 70 | 80 |
| 17 | 23 | 17 | 11 | 3 | 70 | 80 | 65 | 75 |
